# Temperature Increase Enhances *Aedes albopictus* Competence to Transmit Dengue Virus

**DOI:** 10.3389/fmicb.2017.02337

**Published:** 2017-12-01

**Authors:** Zhuanzhuan Liu, Zhenhong Zhang, Zetian Lai, Tengfei Zhou, Zhirong Jia, Jinbao Gu, Kun Wu, Xiao-Guang Chen

**Affiliations:** Department of Pathogen Biology, Guangdong Provincial Key Laboratory of Tropical Disease Research, School of Public Health, Southern Medical University, Guangzhou, China

**Keywords:** *Aedes albopictus*, dengue virus 2, temperature, vector competence, viral titration

## Abstract

Dengue is a mosquito-borne disease that has been an epidemic in China for many years. *Aedes albopictus* is the dominant *Aedes* mosquito species and the main vector of dengue in China. Epidemiologically, dengue mainly occurs in Guangdong Province; it does not occur or rarely occurs in other areas of mainland China. This distribution may be associated with climate, mosquito density, and other factors in different regions; however, the effect of temperature on the vector competence of *Ae. albopictus* for dengue viruses (DENV) remains unclear. In this study, *Ae. albopictus* was orally infected with dengue virus 2 (DENV-2) and reared at constant temperatures (18, 23, 28, and 32°C) and a fluctuating temperature (28–23–18°C). The infection status of the midguts, ovaries, and salivary glands of each mosquito was detected by polymerase chain reaction (PCR) at 0, 5, 10, and 15 days post-infection (dpi). DENV-2 RNA copies from positive tissues were quantified by quantitative real time PCR (qRT-PCR). At 18°C, DENV-2 proliferated slowly in the midgut of *Ae. albopictus*, and the virus could not spread to the salivary glands. At 23 and 28°C, DENV-2 was detected in the ovaries and salivary glands at 10 dpi. The rates of infection, dissemination, population transmission, and DENV-2 copies at 28°C were higher than those at 23°C at any time point. At 32°C, the extrinsic incubation period (EIP) for DENV-2 in *Ae. albopictus* was only 5 dpi, and the vector competence was the highest among all the temperatures. Compared with 28°C, at 28–23–18°C, the positive rate and the amount of DENV-2 in the salivary glands were significantly lower. Therefore, temperature is an important factor affecting the vector competence of *Ae. albopictus* for DENV-2. Within the suitable temperature range, the replication of DENV-2 in *Ae. albopictus* accelerated, and the EIP was shorter with a higher temperature. Our results provide a guide for vector control and an experimental basis for differences in the spatial distribution of dengue cases.

## Introduction

Dengue virus (DENV) is a mosquito-borne flavivirus that can cause a series of diseases, including dengue fever (DF), dengue hemorrhagic fever (DHF), and dengue shock syndrome (DSS) (Guzman and Harris, 2015). It has been estimated that the total number of people infected with DENV is 390 million per year worldwide, of which 96 million showed clinical symptoms and approximately 290 million presented inapparent infection (Bhatt et al., 2013). In China, dengue frequently occurs in the warm southern regions, including Guangdong, Hainan, Guangxi, and Fujian Provinces, while few dengue cases are present in the northern regions (Chen and Liu, 2015). During the period 1990–2015, a total of 73,551 dengue cases were reported in China, and the largest outbreak, with 47,056 cases, caused six deaths in 2014 (Lai et al., 2015; Sun et al., 2017). The number of dengue cases in Guangdong Province accounted for 96% of the national cases in 2014, and 99.8% of these cases were indigenous (Xiao et al., 2016; Zhao et al., 2016). Spatial distribution showed that the highest number of cases was found in Guangzhou City, the capital of Guangzhou Province; few cases were present in Shenzhen City; and no cases were seen in Meizhou City (Xiao et al., 2016). These differences in distribution were probably due to climatic fluctuation, population density, mosquito density, and other factors (Jing et al., 2017).

Temperature is an important factor influencing the ecological habits, vector competence, and extrinsic incubation period (EIP) of mosquitoes for DENV (Xu et al., 2017). Compared to the average temperatures in previous years, the average temperature in Guangdong Province was 0.1–1.3°C higher from July to September in 2014 (Xiao et al., 2016). The maximum average temperature was 30.8°C, and the minimum average temperature was 22.8°C (Xiang et al., 2017). Daily temperatures fluctuated between the highest and lowest temperatures. At high temperatures (≥26°C), transmission of DENV can be observed within 1 week; however, the EIP of DENV is prolonged at low temperatures (≤21°C) (Carrington et al., 2013). When the temperature is below 18°C, DENV cannot spread (Shen et al., 2015).

Dengue viruses are mainly transmitted by *Aedes aegypti* and *Aedes albopictus* (Carneiro and Travassos, 2016). *Ae. albopictus* is the most important dengue vector in China and is widely distributed in the southern region, especially in Guangdong Province (Luo et al., 2015). Guangdong Province has a subtropical climate; the warm and humid conditions are suitable for year-round *Ae. albopictus* breeding. Tires, flower pots, bamboo tubes, and other small containers trapping water are fit for the development of larvae (Li et al., 2014). Adults are active and suck blood many times throughout the day. In this study, based on the average temperature in the Guangzhou region, we collected *Ae. albopictus* mosquitoes from Foshan, Guangdong Province, and used a DENV-2 strain to compare the vector competence of *Ae. albopictus* for DENV-2 under different temperature conditions.

## Materials and Methods

### Mosquitoes

*Aedes albopictus* mosquitoes used in this study have been collected from Foshan, Guangdong Province, China, since 1981. Colony maintenance was conducted under standard insectary conditions (constant 27 ± 1°C, 70–80% relative humidity, and a 16 h:8 h light–dark photoperiod). Eggs in de-chlorinated water were hatched to larvae. One hundred larvae per liter of water were fed turtle food with 0.1 g each day for the first 4 days and then 0.2 g each day until they pupated. Pupae were transferred to cages (20 cm × 20 cm × 35 cm). After emergence, adults were provided with 10% glucose solution or defibrinated sheep blood (Solarbio, Beijing, China) for egg production.

### Dengue Virus

Dengue virus 2 (New Guinea C, GenBank: AF038403.1) was provided by the Key Laboratory of Tropical Disease Control of Sun Yat-sen University (Guangzhou, China). C6/36 cells were cultured in RPMI-1640 medium supplemented with 10% heat-inactivated fetal bovine serum (FBS) and maintained at 28°C. Cells grown in a 75-cm^2^ culture flask were inoculated with DENV-2 at a multiplicity of infection (MOI) of 1. After gentle shaking for 15 min, the culture flask was incubated at 37°C, 5% CO_2_ for 2 days until obvious cytopathic effects. The supernatant was harvested after centrifugation at 1,500 × *g* for 5 min, separated into 0.5-mL aliquots, and frozen at -80°C.

### Viral Titration in C6/36 Cells

Dengue virus 2 titer was determined by 50% tissue culture infective dose (TCID_50_) (Richard et al., 2015). Briefly, C6/36 cells were seeded in a 96-well microtiter plate at a density of 10^5^ cells per well and cultured at 28°C for 24 h. The control solution (RPMI-1640 containing 2% FBS) and 10-fold viral dilutions (10^-1^–10^-11^) were inoculated into C6/36 cells in eight wells. The microtiter plate was placed at 37°C, 5% CO_2_ for 2 h, and then the culture per well was replaced with RPMI-1640 supplemented with 2% FBS. The plate was incubated at 37°C, 5% CO_2_ until the cytopathic effect did not continue to increase. Results were calculated according to the Karber method (Ramakrishnan, 2016).

### Oral Infection with DENV-2

Mosquito infection was conducted in a Biological Safety Level 2 lab. Two days before infection, frozen DENV-2 stock was passaged once more through the C6/36 cells. The titer of the fresh virus was 7.375–7.875 log_10_TCID_50_/mL. The DENV-2 supernatant was collected and mixed with defibrinated sheep blood at a ratio of 2:1. The blood meal was maintained at 37°C for 30 min and transferred into a Hemotek blood reservoir unit (Discovery Workshops, Lancashire, United Kingdom). Five- to seven-day-old female *Ae. albopictus* mosquitoes were glucose starved for 12–24 h and allowed to feed on the infectious blood meal for 30 min. After anesthesia with CO_2_, fully engorged mosquitoes were removed and placed into 250-mL paper cups covered with gauze (10 mosquitoes/cup). Mosquitoes were placed in different HP400GS incubators (Ruihua, Wuhan, China) precisely set at 18, 23, 28, and 32°C. Another group of mosquitoes was placed at the fluctuating temperature (28°C for 14 h, 23°C for 2 h, and 18°C for 8 h). The control group was set for each temperature. DENV-2 stock solution was replaced with RPMI-1640 containing 2% FBS in the control group, the remaining steps were the same as mosquitoes engorged with the infectious blood meal. All treatments were maintained at 80% relative humidity and 16 h:8 h (light:dark) photoperiod, and all mosquitoes were fed 10% glucose solution.

### Vector Competence of *Ae. albopictus* for DENV-2

The midgut, ovaries, and salivary glands of each mosquito from the abovementioned temperatures were dissected and detected at 0, 5, 10, and 15 days post-infection (dpi). The sample size collected from each temperature condition was 3–6 mosquitoes at 0 dpi and 10–16 mosquitoes at each additional time point. The experiment was independently repeated three to five times.

The legs and wings of each mosquito were removed and washed three times in PBS. Disposable insect microneedles were used to separate the midgut, ovaries, and salivary glands of each mosquito under an anatomical lens. Tissues were washed three times in PBS droplets and then transferred to 50 μL of TRIzol (Ambion, Life Technologies, Carlsbad, CA, United States) in 1.5-mL Eppendorf tubes. Total RNA was extracted according to TRIzol manufacturer’s protocol. cDNA was synthesized using a DENV-2-specific primer (5′-TGGTCTTTCCCAGCGTCAAT-3′), and the recommendations of the GoScript^TM^ Reverse Transcription System (Promega, Madison, WI, United States) were followed.

Polymerase chain reaction (PCR) was used to detect DENV-2 in the tissues. A pair of primers was synthesized as described previously (forward primer: 5′-TCAATATGCTGAAACGCGCGAGAAACCG-3′; reverse primer: 5′-TTGCACCAACAGTCAATGTCTTCAGGTTC-3′) (Lanciotti et al., 1992). The target fragment was 511 bp, which was located in the partial capsid and membrane protein region. The total volume of the PCR reaction system was 25 μL, including 12.5 μL Maxima Hot Start Green PCR Master Mix (Thermo Fisher Scientific Inc., Waltham, MA, United States), 0.5 μL of each primer (10 μM), 1 μL cDNA, and 10.5 μL RNase-free water. PCR reaction conditions were the following: 94°C for 3 min, followed by 35 cycles of 94°C for 30 s, 56°C for 30 s and 72°C for 1 min, and 72°C for 7 min. PCR products were identified by 1% agarose gel electrophoresis, ligated with pMD18-T (Takara, Dalian, China) and confirmed by sequencing. Comparing to the tissues from the control group, the positive tissues were determined by detecting specific DENV-2 sequence.

The vector competence of *Ae. albopictus* for DENV-2 transmission was evaluated by infection rate (IR), dissemination rate (DR), and population transmission rate (PTR), as follows (Di Luca et al., 2016):

IR = the number of positive midguts/the total number of midguts
DR = the number of positive ovaries/the number of positive midguts
PTR = the number of positive salivary glands/the total number of tested mosquitoes.

### Quantification of DENV-2 in the Tissues

The amount of DENV-2 in the positive tissues of mosquitoes was further detected by absolute quantitative real-time PCR (qRT-PCR). The plasmid standard was constructed as previously described (Zhang et al., 2010). In brief, the 3′-UTR region of DENV-2 was amplified by PCR using specific primers (forward primer: 5′-TCCCTTACAAATCGCAGCAAC-3′; reverse primer: 5′-TGGTCTTTCCCAGCGTCAAT-3′). The fragment with 127 bp was cloned into pMD18-T and linearized by *EcoR*I. The concentration of the plasmid was 26.9 ng/μL, which was transformed into a copy number (8.71 × 10^9^/μL).

The qRT-PCR reaction mixture per well contained 10 μL SYBR^®^ selected master mix, 1 μL of each primer (10 μM), 2 μL cDNA or the plasmid standard, and 6 μL RNase-free water. The reaction was performed in the 7500 Real-Time PCR System as follows: 50°C for 2 min, 95°C for 2 min, followed by 40 cycles of 95°C for 15 s, 60°C for 15 s, and 72°C for 1 min. Melting curves were given at 95°C for 15 s, 60°C for 1 min, 95°C for 30 s, and 60°C for 15 s. A standard curve was established by 10-fold dilutions of the plasmid standard (8.71 × 10^2^–8.71 × 10^7^). The result of qRT-PCR was ascertained by using non-template, negative (mosquito infected with C6/36 cells) and positive control (mosquito infected with DENV-2 at 0 dpi). Its minimum detecting amount is 87.1 copies/reaction of DENV-2. Each sample was conducted in three replicates, and the results were determined by the melting curve and cycle threshold values.

### Data Analysis

All statistical analyses were performed with SPSS 20.0 (IBM, Chicago, IL, United States). Under constant temperature, the vector competence of *Ae. albopictus* for DENV-2 was analyzed using logistic regression. IR, DR, and PTR were separately compared at different temperatures or different time points. *P*-value was corrected by Bonferroni adjustments. Chi-square (and Fisher’s exact) tests were used to determine the difference in vector competence between 28°C and the fluctuating temperature (28-23-18°C). The amounts of DENV-2 in the tissues were log-transformed and then analyzed by *post hoc* Tukey’s LSD tests of variance analysis for the constant-temperature groups. Student’s *t*-test was used to compare the DENV-2 titer of the tissues between 28°C and (28-23-18°C). *P* < 0.05 was considered statistically significant.

## Results

### Temperature Affects Vector Competence of *Ae. albopictus* for DENV-2

#### Constant Temperatures

**FIGURE 1:**
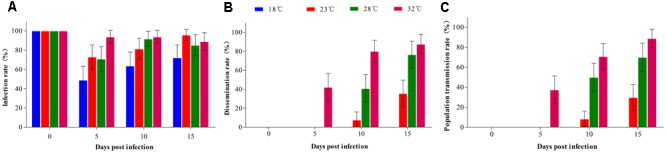
Vector competence of *Ae. albopictus* orally infected with DENV-2 at different temperatures. *Ae. albopictus* orally infected with DENV-2 were reared at 18, 23, 28, and 32°C. The midguts, ovaries, and salivary glands of mosquitoes reared at each temperature were dissected at 0, 5, 10, and 15 days post-infection and detected by PCR. **(A)** Infection rate (IR): no. positive midguts/total no. midguts; **(B)** Dissemination rate (DR): no. positive ovaries/no. positive midguts; **(C)** Population transmission rate (PTR): no. positive salivary glands/total no. mosquitoes. Error bars represent 95% confidence interval (CI).

In this study, a total of 602 *Ae. albopictus* females reared at 18, 23, 28, and 32°C were used to measure the rates of infection, dissemination, and transmission of DENV-2. At 0 dpi, the IRs of mosquitoes were 100% at all temperatures, indicating that all mosquitoes ingested the blood meal containing DENV-2 (**Figure 1A**). After the infectious blood meal was digested, the IRs of *Ae. albopictus* at 18, 23, and 28°C were significantly reduced at 5 dpi compared with 0 dpi (*z* = 10.329, *P* = 0.001; *z* = 4.149, *P* = 0.0042; *z* = 4.565, *P* = 0.003) and then gradually increased at 10 and 15 dpi (**Figure 1A**). However, *Ae. albopictus* maintained an invariably high IR at 32°C, and no significant difference was present at any time point (**Figure 1A**). At 5 dpi, the IR of *Ae. albopictus* was obviously lower at 18°C than that at the other three temperatures (*z* = 8.991, *P* = 0.003; *z* = 7.538, *P* = 0.006; *z* = 17.448, *P* < 0.001). IRs at 23 and 28°C showed no difference; however, they were lower than the IR at 32°C (*z* = 6.048, *P* = 0.014; *z* = 6.933, *P* = 0.008). At 10 and 15 dpi, IRs at 23, 28, and 32°C were not significantly different; however, they were higher than the IRs at 18°C (*z* = 15.374, 21.725, 9.975, *P* < 0.01; *z* = 20.990, 15.110, 9.563, *P* < 0.01).

Dengue virus 2 was disseminated to the ovaries as early as 5 dpi at 32°C; however, the DR was low at this point. DRs at 10 and 15 dpi were significantly higher than those at 5 dpi (*z* = 12.085, 15.546, *P* < 0.01), and no difference occurred between 10 and 15 dpi (**Figure 1B**). At 23 and 28°C, DENV-2 detected in the ovaries started 10 dpi, and the DR at 15 dpi was higher than that at 10 dpi (*z* = 7.434, *P* = 0.006; *z* = 8.841, *P* = 0.003). At 10 dpi, the DR arranged in descending order was 32, 28, and 23°C, and there were significant differences between any two temperatures (**Figure 1B**). At 15 dpi, the DR was still lower at 23°C than that at 28°C (*z* = 12.659, *P* < 0.00) and 32°C (*z* = 19.429, *P* < 0.001); however, no difference appeared between 28 and 32°C (z = 1.402, *P* = 0.236). No positive ovaries were detected at 18°C during the experiment (**Figure 1B**).

The infection status in the salivary glands was similar to that in the ovaries. Detection of DENV-2 in the salivary glands was 5 dpi at 32°C and 10 dpi at 23 and 28°C, and the PTR increased gradually over time (**Figure 1C**). At 10 dpi, the PTR was lower at 23°C than those at 28 and 32°C (*z* = 15.974, 29.198, *P* < 0.001), and the PTR at 28°C was lower than that at 32°C (*z* = 4.097, *P* = 0.045). Similarly, the PTR at 15 dpi was highest at 32°C among all temperatures. At 18°C, DENV-2 in the salivary glands was negative at all time points of the experiment (**Figure 1C**).

#### Fluctuating Temperature

**FIGURE 2:**
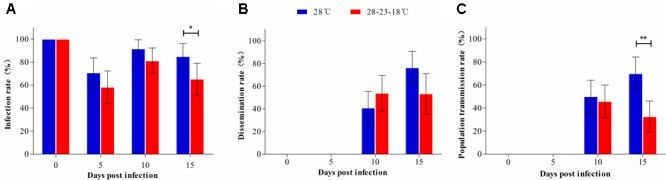
Comparison of vector competence of *Ae. albopictus* between constant and fluctuating temperatures. The IR, DR, and PTR of *Ae. albopictus* reared at 28°C and 28–23–18°C were compared at 0, 5, 10, and 15 dpi. **(A)** IR; **(B)** DR; **(C)** PTR. Error bars represent 95% CI. ^∗^*P* < 0.05; ^∗∗^*P* < 0.01.

We separately compared the IR, DR, and PTR of *Ae. albopictus* reared under constant (28°C) and fluctuating temperature (28–23–18°C) conditions. IRs at 0, 5, and 10 dpi showed no significant differences between 28 and 28–23–18°C; however, the IR at 28°C was higher than that at 28–23–18°C at 15 dpi (χ^2^ = 4.398, *P* = 0.048) (**Figure 2A**). Whether *Ae. albopictus* was reared at 28 or 28–23–18°C, positive ovaries and salivary glands were detected at 10 dpi. The DR at 10 and 15 dpi showed no difference between mosquitoes from constant and fluctuating temperature conditions (**Figure 2B**). At 10 dpi, the PTR at 28°C was the same as that at 28–23–18°C. However, the PTR at 28°C was significantly higher than that at 28–23–18°C at 15 dpi (χ^2^ = 11.965, *P* = 0.001) (**Figure 2C**).

### Temperature Affects the Amount of DENV-2 in the Tissues of *Ae. albopictus*

#### Constant Temperatures

**FIGURE 3:**
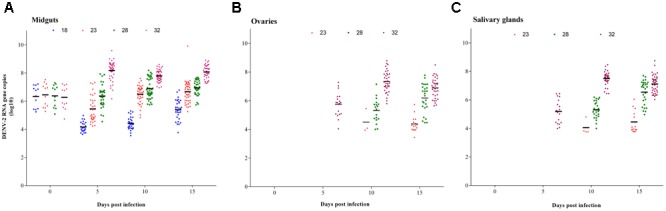
DENV-2 RNA copies in tissues of *Ae. albopictus* at the constant temperature. The amount of DENV-2 in the positive tissues of *Ae. albopictus* was further detected by qRT-PCR. **(A)** Midguts; **(B)** ovaries; **(C)** salivary glands. Horizontal black lines show median DENV-2 copies (log_10_) at each temperature.

The amounts of DENV-2 in the midguts, ovaries, and salivary glands of *Ae. albopictus* were further detected by qRT-PCR. At 0 dpi, DENV-2 titer showed no significant differences among all temperature groups (**Figure 3A**). At 18°C, DENV-2 copies (log_10_) of the midguts decreased markedly from 0 (6.33 ± 0.77) to 5 dpi (4.17 ± 0.37) and then increased slowly over time. Until 15 dpi, the level of DENV-2 was still lower than at 0 dpi (*P* < 0.05) (**Figure 3A**). Similarly, DENV-2 of the midguts at 23°C was lower at 5 dpi than that at 0 dpi, followed by a rapid recovery. The amount of DENV-2 at 10 dpi was close to that at 0 dpi, and no difference was found between 15 and 10 dpi (*P* > 0.05). At 28°C, DENV-2 showed a slow increasing trend in the midguts of *Ae. Albopictus* mosquitoes. DENV-2 copies (log_10_) at 10 and 15 dpi were higher than those at 0 and 5 dpi (*P* < 0.05); however, there was no difference between 0 and 5 dpi or between 10 and 15 dpi (*P* > 0.05). At 32°C, DENV-2 copies in the midguts increased rapidly to 8.18 ± 0.66 (log_10_) before 5 dpi and then trended toward stability (**Figure 3A**).

Dengue virus 2 detected in the ovaries was the earliest at 32°C (**Figure 3B**). The viral titration (log_10_ copies) was 5.75 ± 0.83 at 5 dpi, and reached its peak at 10 dpi (7.34 ± 0.74). There was no significant difference between 10 and 15 dpi (*P* > 0.05). DENV-2 in the ovaries at 23 and 28°C started from 10 dpi. DENV-2 copies were lower at 23°C than those at 28°C; these values were significantly lower than those at 32°C by this time (*P* < 0.05) (**Figure 3B**). After that, the virus in the ovaries at 28°C increased quickly from 10 to 15 dpi; however, it remained stable at 23°C (**Figure 3B**). The variation trend of DENV-2 titer in the salivary glands was the same as in the ovaries (**Figure 3C**). DENV-2 copies (log_10_) in the salivary glands at 32°C were higher than those at 23 and 28°C at any time point, and they were higher at 28°C than those at 23°C (**Figure 3C**). DENV-2 was not detected in the ovaries or salivary glands at 18°C (**Figures 3B,C**).

#### Fluctuating Temperature

**FIGURE 4:**
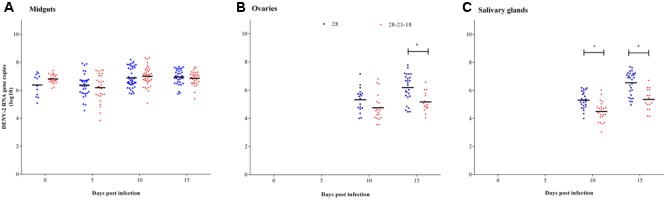
Comparison of DENV-2 RNA copies in tissues of *Ae. albopictus* between constant and fluctuating temperatures. The amount of DENV-2 in tissues of *Ae. albopictus* reared at 28°C and 28–23–18°C was compared at 0, 5, 10, and 15 dpi. **(A)** Midguts; **(B)** ovaries; **(C)** salivary glands. Horizontal black lines show median DENV-2 copies (log_10_) at each temperature. ^∗^*P* < 0.05.

The amounts of DENV-2 in the midguts, ovaries, and salivary glands of *Ae. albopictus* mosquitoes reared at 28 and 28–23–18°C were compared at 0, 5, 10, and 15 dpi. In the midguts, the DENV-2 copy number showed no difference between constant and fluctuating temperature groups at any time point (*P* > 0.05) (**Figure 4A**). In the ovaries, the DENV-2 titer at 28 and 28–23–18°C showed no significant difference at 10 dpi, although it was higher at 28°C than that at 28–23–18°C at 15 dpi (*P* < 0.05) (**Figure 4B**). In the salivary glands, DENV-2 copies (log_10_) at 28°C were obviously higher than those at 28–23–18°C at 10 and 15 dpi (*P* < 0.05) (**Figure 4C**).

## Discussion

In recent years, the epidemic area of dengue has been expanding with the acceleration of climate warming, trade globalization, and urbanization (Castro et al., 2017). Mosquito control is an effective measure to prevent a dengue outbreak (Pang et al., 2017). Clarifying the vector competence and EIP of *Ae. albopictus* for the DENV under different temperatures would provide guidance for vector control. Our results showed that DENV-2 was confined to the midguts of *Ae. albopictus* at 18°C and could invade the salivary glands between 23 and 32°C. Shorter EIPs and higher transmission rates were detected with higher temperatures.

The lower critical value under which mosquitoes could not spread the virus was reported to be 18°C (Shen et al., 2015). In our study, DENV-2 rapidly decreased within 5 days and then slowly increased. DENV-2 could not break through the midgut barrier to spread to the salivary glands during the experimental time. If sufficient time was given, it would be possible to infect the ovaries and the salivary glands. However, Xiao et al. (2014) demonstrated that DENV was still not detected in the salivary glands when the experimental time was extended to 25 dpi.

The EIP at 23 and 28°C was consistent (10 dpi). However, the IR, TR, and PTR of *Ae. albopictus* mosquitoes and the amount of DENV-2 in the tissues were higher at 28 than those at 23°C except for 0 dpi. The titer of DENV was a key factor affecting mosquito infection. When the log_10_ plasma viremia level of DENV was between 4 and 10, the susceptibility of *Aedes* was enhanced with the increase of virus titer, and *Ae. aegypti* and *Ae. albopictus* could not be infected when the log_10_ plasma viremia level was below 4 (Whitehorn et al., 2015). At 23°C, the viral amount in the ovaries and salivary glands of *Ae. albopictus* was low (4–4.5 log_10_ copies/mL). Whether DENV-2 can be transmitted through mosquito bites requires further study.

The EIP of DENV-2 in *Ae. albopictus* was gradually shortened with rising temperature; therefore, more hosts would be infected by *Ae. albopictus* bites in the limited reproductive cycle. At 32°C, DENV-2 could spread to the ovaries and salivary glands of *Ae. albopictus* by 5 dpi. The annual average temperature was higher than 30°C from July to October in Guangzhou, and the high density of *Ae. albopictus* and the short EIP caused an outbreak of dengue during this season. In this study, the upper temperature limit was set at 32°C because pre-experimental results showed that the mortality rate of *Ae. albopictus* was extremely high and the mosquitoes would not survive until the end of the experiment.

In addition, we compared the vector competence and DENV-2 titer of *Ae. albopictus* reared at constant (28°C) and fluctuating (28–23–18°C) temperatures. The results showed that, compared with the amount of DENV-2 in the salivary glands at 28°C, the amount of DENV-2 in the salivary glands at 28–23–18°C was significantly decreased. In China, dengue cases occur mainly in warm southern cities, and few cases are present in northern cities. In addition, Guangzhou and Shenzhen are both economically developed, densely populated cities in Guangdong Province; however, the number of cases of dengue was very different in 2014: with 96% of cases concentrated in Guangzhou. We speculated that diurnal temperature ranges in northern cities and Shenzhen belonging to coastal areas were large. These fluctuations affected the proliferation and spread of the DENV in mosquitoes.

Although we used a single serotype (DENV-2) to detect the vector competence of *Ae. albopictus* at different temperatures, a previous study showed that the vector competence of *Ae. aegypti* for different serotypes of DENV (DENV-1 or DENV-2) was similar under similar temperature conditions (Lambrechts et al., 2011). In addition, the survival rate of *Ae. albopictus* was not considered in this study because it is extremely high in nature, and even a few surviving mosquitoes could transmit DENV to people. To ensure the parallelism of the experiment, adapted mosquitoes in the laboratory were used. It is possible that if first generation lab mosquitoes were to be used, results in this study might be different.

## Author Contributions

ZhL and X-GC designed the study. ZhL, ZZ, ZeL, TZ, ZJ, JG, and KW carried out data acquisition and analysis. ZeL wrote the paper. X-GC supervised the study. All authors reviewed the manuscript.

## Conflict of Interest Statement

The authors declare that the research was conducted in the absence of any commercial or financial relationships that could be construed as a potential conflict of interest. The reviewer AM and handling Editor declared their shared affiliation
